# Somatosensory phenotyping in women with persistent genital arousal disorder: a case–control study

**DOI:** 10.1097/PR9.0000000000001509

**Published:** 2026-10-16

**Authors:** Janneke van Uhm, Astrid Hamel, Albert Dahan, Marieke Niesters, Maxime Kummeling, Stephanie Both, Henk Elzevier, Monique van Velzen

**Affiliations:** a Department of Urology, Leiden University Medical Center, Leiden, the Netherlands; b Department of Anesthesiology, Leiden University Medical Center, Leiden, the Netherlands; c MediD Consultancy Group, Amsterdam, the Netherlands; d Outcomes Research Consortium, Houston, TX, USA; e Centre for Human Drug Research, Leiden, the Netherlands; f Department of Anesthesiology, Erasmus Medical Center, Rotterdam, the Netherlands; g Department of Sexology and Psychosomatic Gynecology, Amsterdam UMC, Amsterdam, the Netherlands

**Keywords:** Persistent genital arousal disorder, Quantitative sensory testing, Conditioned pain modulation, Phenotyping

## Abstract

**Introduction:**

Persistent genital arousal disorder (PGAD) is a rare and distressing condition characterized by persistent, unwanted genital arousal in the absence of sexual desire or stimulation. Persistent genital arousal disorder is increasingly recognized as a disorder of abnormal genital sensory processing, but objective neurophysiological data remain limited.

**Methods:**

We conducted an exploratory case–control study to characterize somatosensory function in women with PGAD. Thirty women with PGAD and 30 age-matched healthy controls were recruited at Leiden University Medical Center. Participants underwent quantitative sensory testing (QST) at hand and external genital sites, conditioned pain modulation, and corneal confocal microscopy.

**Results:**

Hand QST values were within normal range in both groups. In the genital region, PGAD participants showed altered thermal pain processing, with cold and heat hyperalgesia compared with controls. Mechanical pain thresholds did not differ between groups; however, PGAD participants reported higher pain intensity ratings to mechanical stimuli and showed significantly greater temporal summation of pain in genital regions. Genital dynamic mechanical allodynia occurred more frequently in PGAD participants but did not differ significantly at the group level. Conditioned pain modulation magnitude did not differ between groups and showed substantial interindividual variability. Corneal confocal microscopy abnormalities were observed in a proportion of participants in both groups without significant between-group differences.

**Discussion:**

These findings show that QST is feasible in women with PGAD and indicate that PGAD is associated with altered somatosensory processing demonstrating genital thermal hypersensitivity and central pain sensitization in a subset of patients.

## Abstract 2

Women with persistent genital arousal disorder exhibit heterogeneous alterations in genital somatosensory processing, with thermal hyperalgesia and signs of central pain sensitization, indicating a variable neuropathological phenotype.

## 1. Introduction

Persistent genital arousal disorder (PGAD) is a rare and distressing condition characterized by persistent, involuntary genital arousal in the absence of sexual desire or stimulation. Symptoms are typically intrusive, unwanted, and are often not relieved by orgasm. Persistent genital arousal disorder is currently defined by 5 diagnostic criteria: (1) persistent, involuntary genital arousal; (2) persistent physical genital arousal following one or more orgasms; (3) absent relationship between genital arousal and subjective sexual desire; (4) arousal sensations experienced as intrusive and unwanted; and (5) significant distress associated with symptoms.^15^ Persistent genital arousal disorder is reported predominantly in women, although increasing numbers of case reports describe PGAD-like symptoms in men, including unpleasant genital sensations and spontaneous ejaculations.^24,30^ In affected women, symptoms frequently manifest as genitopelvic dysesthesias localized to the clitoris but may also involve vulva, urethra, and perineum. Beyond physical symptoms, PGAD is often associated with substantial psychological burden, including anxiety, depression, social withdrawal, and, in severe cases, suicidal ideation.^5^ Persistent genital arousal disorder is increasingly recognized as a disorder with abnormal genital sensory processing rather than solely a disorder of sexual dysfunction.

Despite growing recognition, the underlying mechanisms of PGAD remain incompletely understood. Evidence suggests that PGAD may result from a combination of neurological, vascular, pharmacological, and psychological factors.^3,9,12,31^ Patients commonly report sensory disturbances characteristic of neuropathic disorders. Cases have been associated with pudendal nerve dysfunction, which is essential for genital sensation and arousal, and structural abnormalities such as Tarlov cysts.^19^ Comorbidity with restless legs syndrome and overactive bladder further supports a neurological contribution.^14,28^ Onset has been linked to initiation or discontinuation of selective serotonin reuptake inhibitors.^13^ Psychological factors, including anxiety and obsessive-compulsive traits, may exacerbate symptom perception and distress.^10^

Patients with PGAD frequently report hypersensitivity to touch or pressure in the genital region, for example, from clothing or seating, as well as dysesthetic sensations such as tingling, burning, and twitching pain. These symptoms closely resemble neuropathic phenomena and suggest abnormalities in genital somatosensory processing. They may reflect altered peripheral nerve function, including small fiber neuropathy.^19,31^ Objective evidence supporting these underlying mechanisms remains limited.

We performed an exploratory study to further characterize somatosensory function in PGAD. We applied quantitative sensory testing (QST) at genital and extragenital sites, evaluated endogenous pain inhibition using conditioned pain modulation (CPM), and assessed small nerve fiber morphology using corneal confocal microscopy (CCM). Women with PGAD were compared with healthy female controls.

## 2. Methods

### 2.1. Ethics and registration

This single-center case-control study was conducted at the Anesthesia and Pain Research Unit of the Department of Anesthesiology and Urology at Leiden University Medical Center (Leiden, the Netherlands). The study protocol was approved by the Medical Research Ethics Committee (MREC Leiden the Hague Delft, the Netherlands). All participants provided written and oral informed consent before enrollment. The study was preregistered at the OMON register of the Dutch Competent Authority (https://onderzoekmetmensen.nl) under identifier NL-OMON52300 on April 8, 2021. The study was conducted between June 2021 and November 2023. All procedures complied with the Declaration of Helsinki and Good Clinical Practice guidelines.

### 2.2. Participants

Participants with PGAD were recruited through referrals from their urologist, whereas healthy controls were recruited via paper flyers distributed in public locations. Inclusion criteria included the following: age ≥18 years, female, and able to provide written informed consent. Participants were eligible for the PGAD group if they had a formal PGAD diagnosis in accordance with the ISSWSH consensus panel.^5^ Healthy controls were eligible if they had no diagnosis of a pain syndrome, reported an average daily pain score <2 on an 11-point numerical rating scale (NRS; range 0–10, where 0 indicates no pain and 10 worst pain imaginable), and had no overactive bladder symptoms, urinary complaints, or sexual problems at the time of screening. Mild and infrequent pain in controls was accepted (eg, menstrual pain, occasional mild headache). Persistent genital arousal disorder participants and healthy controls were excluded if they reported the following: (1) regular opioid use (>3/7 days), (2) diagnosis of a central nervous system disease, or (3) inability to undergo QST in the genital region based on judgment of participant or investigator. Nociplastic pain conditions and functional neurological disorders were not diagnostically assessed or excluded.

### 2.3. Study design

Verbal and written study information was given to potential participants, who were given sufficient time to consider participation and ask questions. Interested individuals scheduled a visit to the research unit according to their availability. The study visit included provision of written informed consent and eligibility screening. Participants meeting eligibility criteria proceeded directly to experimental measurements. During the visit, participants completed questionnaires and underwent measurements. All participants received monetary compensation and travel expense reimbursement.

### 2.4. Questionnaires

Medical history was taken by standardized questionnaires by both groups, which included items on current or past conditions such as pain, depression, restless legs, urinary frequency, and menopause. Medication use was self-reported during study visit and documented by the investigator. Participants with PGAD completed questionnaires addressing duration, onset, localization of PGAD symptoms, and previously received treatments.

### 2.5. Measurements

Participants underwent a physical examination including vital signs and measurements of height and weight. Subsequently, they underwent measurements described below allowing ample resting time between tests. Adverse events were recorded for each participant from the time of informed consent until 24 hours after completion of study procedures.

#### 2.5.1. Quantitative sensory testing

Quantitative sensory testing was performed by 3 trained investigators using standardized instructions. All measurements were based on the protocol of the German Research Network on Neuropathic pain (DFNS), with minor adjustments for genital testing locations due to anatomical constraints and to limit testing duration.^23^ Thermal and mechanical sensory testing was performed at multiple anatomical sites. The hand served as control location. For the external genital region, 3 mapping sites were selected bilaterally (6 locations in total) based on vulvar innervation by pudendal and ilioinguinal nerves and previous genital sensory testing studies (Fig. 1).^21,31^ Because normative values for genital testing are unavailable, healthy controls served as interindividual comparators. Hand testing was performed using the 3 × 3 cm thermode attached to a TSA2 thermosensory stimulator (Medoc Ltd, Israel), while participants were seated. Genital thermal testing was performed using the 6-mm Tiny Thermode (Medoc Ltd) with participants in lithotomy position. Cold detection thresholds were determined using 3 measurements identifying the temperature at which cold was first perceived. Baseline temperature was 32°C and decreased to 0°C (1°C/s). Warm detection thresholds were obtained similarly, with temperatures increasing from 32°C to 50°C (1°C/s). Thermal sensory limen and paradoxical thermal sensations (PTS) were assessed using alternating warm and cold stimuli. Participants indicated the perceived sensation, and PTS were scored dichotomously by the investigator. Cold and heat pain thresholds were assessed similarly, with participants indicating when stimuli became painful. Three measurements were obtained and averaged for all thermal tests.

**Figure 1.**
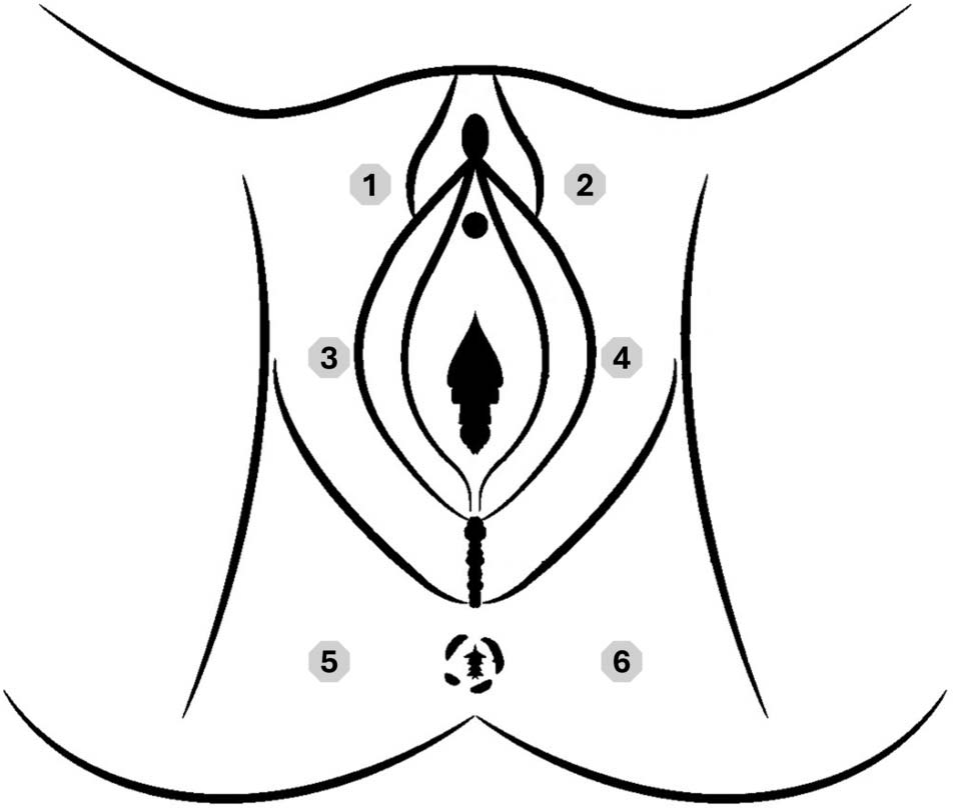
Quantitative sensory testing locations of the genital area. Lateral locations: 1 and 2, clitoris; 3 and 4, labium majora; and 5 and 6, at the level of ischial tuberosity.

Mechanical pain thresholds were determined using 7 weighted PinPrick stimulators (8–512 mN, MRC-Systems, Germany). Each stimulus was applied twice, and participants indicated whether the sensation was dull or sharp. Five dull and 5 sharp responses were averaged to obtain mechanical pain thresholds. Mechanical pain sensitivity was assessed using the same stimulators in semirandomized order. Participants rated stimulus intensity (NRS), and mean scores were calculated. Dynamic mechanical allodynia (ALL) was assessed using cotton wisp, cotton tip on elastic strip, and brush. Participants rated painfulness (NRS). Temporal summation (wind-up) was assessed using the PinPrick stimulator that elicited an NRS of approximately 1 during mechanical pain sensitivity testing. At each location, participants rated NRS after a single stimulus and after a train of 10 stimuli (1 Hz).

#### 2.5.2. Conditioned pain modulation

Central pain inhibition was assessed using a previous CPM paradigm.^17^ The test stimulus consisted of a 10-second heat stimulus applied to the volar side of the nondominant forearm using the 3 × 3 cm thermode (TSA2, Medoc Ltd). The temperature was individually calibrated to elicit a peak pain score of approximately 60 mm on an electronic visual analog scale (CoVAS, Medoc Ltd). The conditioning stimulus consisted of cold-water immersion of the contralateral foot (6°C), and participants rated its painfulness (NRS). Participants rated perceived pain intensity of the heat stimulus using CoVAS slider. Test and conditioned stimuli were repeated 3 times, and the results were averaged. To prevent sensitization, thermode position was slightly adjusted between trials with sufficient intervals between stimuli.

#### 2.5.3. Corneal confocal microscopy

Bilateral CCM was performed using the Rostock Cornea Module of the Heidelberg Retina Tomograph III (Heidelberg Engineering, Germany). After topical anesthesia with oxybuprocaine (Théa Pharma BV) and lubrication with Vidisic Carbogel (Bausch & Lomb Pharma), the microscope objective was positioned at the corneal apex. Images of corneal nerve plexi were acquired with a field of view of 400 × 400 µm and analyzed using ACCmetrics software (University of Manchester, United Kingdom). Corneal nerve fiber length, nerve fiber density (CNFD), and nerve branching density (CNBD) were quantified after manual selection (participant-blinded) of 3 to 8 representative high-quality images per eye. Values were compared with published reference values.^26^ Small nerve fiber pathology was defined as abnormal values in at least 2 of 3 parameters.

### 2.6. Sample size and statistical analysis

This exploratory case–control study used a convenience sample based on feasibility considerations. The target sample size was consistent with previous studies in comparable populations.^2,20,29^ We aimed to include 30 women with PGAD and 30 age-decade–matched healthy controls.

Statistical analyses were performed using IBM SPSS Statistics v28. Statistical significance was set at *P* < 0.05. Group differences in participant characteristics (age and body mass index [BMI]) were assessed using independent *t*-tests. Associations between categorical variables were analyzed using the Chi-square tests or Fisher exact tests when expected cell counts were <5. Hand QST values were transformed to *Z*-scores,^23^ whereas genital QST measurements are presented as raw values. Thermal hyperalgesia, defined as detection-to-pain interval, was calculated as the difference between thermal pain and detection thresholds. Between-group differences in continuous QST parameters were analyzed using independent *t*-tests. Paradoxical thermal sensations and allodynia were analyzed dichotomously using the Fisher exact tests and Mann–Whitney *U*-tests.

Conditioned pain modulation magnitude was calculated as the percentage reduction in peak pain score: CPM (%) = 100 × [(NRS without conditioning stimulus−NRS with conditioning stimulus)/NRS without conditioning stimulus]. Triplicates were averaged, and between-group differences were assessed using independent *t*-tests.

For CCM parameters, descriptive statistics were calculated, and groups were compared using independent *t*-tests. Normality was assessed using the Shapiro–Wilk test and visual inspection of Q-Q plots.

## 3. Results

### 3.1. Participant characteristics

Thirty-six patients with PGAD were invited. Five declined participation and one was excluded due to vulvar lichen sclerosis. Among healthy volunteers, 90 received study information or were assessed for eligibility; 57 declined participation or did not respond to follow-up contact. At screening, 2 healthy volunteers were excluded due to pain levels caused by cervical hernia and fibromyalgia. One volunteer was excluded because reliable QST responses could not be obtained. In total, 30 PGAD participants and 30 age-decade–matched healthy controls were included for analyses. The mean age of PGAD participants was 53.7 ± 12.4 years (mean ± SD, range 30–74 years), and the mean BMI was 24.2 ± 4.9 kg/m^2^ (16–40 kg/m^2^). Age and BMI did not differ between PGAD participants and controls (*P* > 0.05). Premenopausal and postmenopausal state was comparable between groups. Participants with persistent genital arousal disorder reported significantly more symptoms and comorbidities and more pain than controls and used medication more frequently (Table 1).

**Table 1:** General characteristics of persistent genital arousal disorder participants and healthy controls.

	PGAD	Controls	*P*
	(N = 30)	(N = 30)	
General characteristics			
Age in years (range)	53.7 ± 12.4 (30–74)	48.1 ± 15.7 (19–79)	0.13
BMI (kg/m^2^)	24.7 ± 4.9 (16–40)	24.3 ± 3.5 (18–32)	0.77
Hormonal status*			0.59
Premenopausal	14	13	
Postmenopausal	11	14	
Other, undefined	5	3	
Symptoms and diseases			
Average pain intensity (past 4 wks)			<0.0001
No pain	4	19	
Mild pain	10	10	
Moderate pain	14	0	
Severe pain	2	0	
Neuropathic pain	12	0	
Depression	16	2	<0.001
Anxiety	11	1	0.001
Restless legs	12	2	0.002
Constipation	13	1	<0.001
Urinary frequency	18	3	<0.001
Urinary tract infections	15	7	0.03
Medication use			
Antidepressants (amitriptyline, clomipramine, fluoxetine, and sertraline)	5	0	
Antiepileptics (clonazepam and gabapentin)	7	0	
Benzodiazepine (oxazepam, diazepam, and lorazepam)	4	0	
Analgesics (paracetamol and meloxicam)	2	0	
Others (eg, antiplatelet agents and proton-pump inhibitors)	9	8	1.000

Age and body mass index (BMI) are presented as means ± SD (range). Pain intensity is scored as average pain categorical qualification in the past 4 wks. The 7 most frequently reported symptoms and comorbid conditions are shown as number of participants. Medication use at the time of the study visit is reported as number of participants. A *P*-value of < 0.05 was considered statistically significant.

* Premenopausal, menses < 1 y; postmenopausal, menses > 1 y; other, undefined due to oral anticonception, hormonal intrauterine device, or hysterectomy.

BMI, body mass index; PGAD, persistent genital arousal disorder.

### 3.2. Persistent genital arousal disorder symptoms

The median self-reported duration of unwanted genital sensations was 5.1 years (Interquartile Range [IQR] 1.8–7.4 years, range 0.5–54.1 years). Clinical characteristics of PGAD are summarized in Table 2. More than half of the participants reported a sudden onset of symptoms (56.7%), 33.7% described a gradual onset, and the remaining participants were unable to recall the onset. Over time, most patients reported either an increase (46.7%) or fluctuations in severity (46.7%), with a smaller proportion (6.7%) indicating a decrease in severity. Symptoms were reported across multiple sites within the genital and lower abdominal regions. The clitoris was affected in 80% of participants, and most participants reported sensations in more than one location. The most commonly reported triggers were pressure on the genital area (eg, clothing, seating, or bicycle saddles) (80.0%), psychological stress (70.0%), micturition (56.7%), and vibrations from external sources such as vehicles (56.7%). Distraction (73.3%) and physical activity (46.7%) were the most frequently reported factors that alleviated PGAD symptoms.

**Table 2:** Symptoms and treatment history of persistent genital arousal disorder participants.

PGAD characteristics	n (%)
Onset of sensations	
Suddenly	17 (56.7)
Gradually	11 (36.7)
Unknown	2 (6.7)
Severity over time	
Increased	14 (46.7)
Unchanged	0 (0.0)
Decreased	2 (6.7)
Varying	14 (46.7)
Location PGAD sensations*	
Labia majora	8 (26.7)
Labia minora	13 (43.4)
Clitoris	24 (80.0)
Introitus	11 (36.7)
Deep in the vagina	6 (20.0)
Inside of thighs	3 (10.0)
Lower abdomen	9 (30.0)
Radiation of sensations	19 (63.3)
Medication used for PGAD symptoms	15 (50.0)
Previous treatments of PGAD symptoms	25 (83.3)
Treatment of PGAD symptoms by*	
Gynecologist	14 (46.7)
Urologist	9 (30.0)
Psychiatrist or psychologist	11 (36.7)
Sexologist	6 (20.0)
(Pelvic) physiotherapist	9 (16.7)
Pain medicine physician	3 (10.0)

Data are presented as number and percentage (%) of the total sample (n = 30).

* Multiple options are possible.

PGAD, persistent genital arousal disorder.

### 3.3. Quantitative sensory testing

Hand QST values were compared with published reference values (Fig. 2). On average, neither PGAD participants nor controls showed abnormal thermal detection thresholds, thermal pain thresholds, or mechanical pain responses at the hand. Paradoxical thermal sensations were observed in 1 control participant (3.3%) and 2 PGAD participants (6.7%). Mechanical allodynia was present in 3 PGAD participants (10%) and in none of the controls.

**Figure 2.**
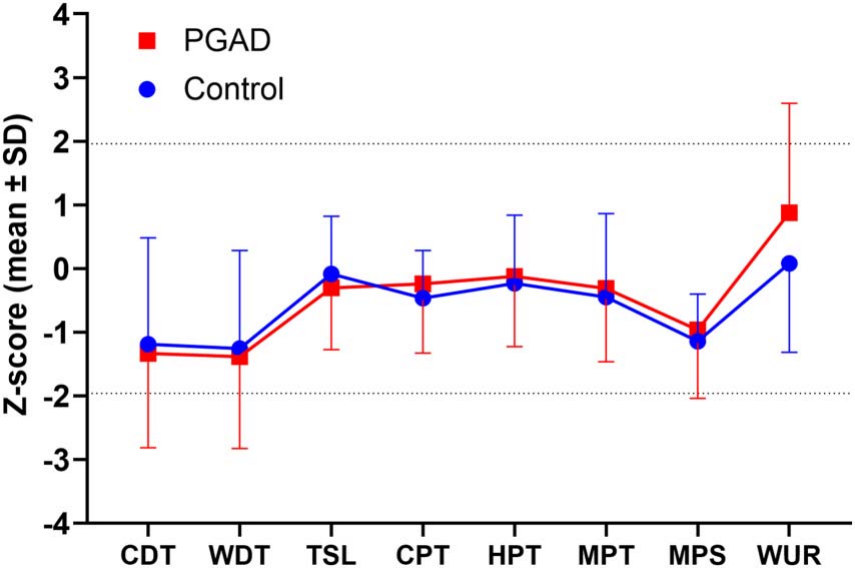
Quantitative sensory testing *Z*-scores on the hand location of PGAD participants (red squares) and controls (blue circles). Symbols represent means, and error bars are SDs. Horizontal dashed lines represent the 1.96 bars for SD from normal (*Z*-score of 0). On the *x*-axis, tests are listed where abbreviations are CDT, cold detection threshold; CPT, cold pain threshold; HPT, heat pain threshold; MPS, mechanical pain sensitivity; MPT, mechanical pain threshold; PGAD, persistent genital arousal disorder; TSL, thermal sensory limen; WDT, warm detection threshold; WUR, wind-up ratio.

Because normative values are unavailable for genital testing, the results are presented as raw values (Table 3 and Fig. 3). Cold detection thresholds were numerically lower in PGAD participants compared with controls across locations, although not statistically significant (*P* = 0.40) (Fig. 3A). Warm detection thresholds were similar between groups (*P* = 0.93) (Fig. 3B). Thermal sensory limen was significantly lowered in PGAD patients, compared with controls (patients 29.7 ± 1.1°C, controls 30.9 ± 0.9°C, difference 1.28 ± 0.6°C, *P* = 0.05, Fig. 3C). Paradoxical thermal sensations were infrequent and did not differ between groups (*P* > 0.99) (Fig. 3D).

**Table 3:** Results of quantitative sensory testing for genital locations.

Test	Control	PGAD	Mean difference (95% CI)	*P*
CDT (°C)	21.6 ± 3.5	19.9 ± 3.3	1.72 (−2.65 to 6.08)	0.40
WDT (°C)	38.7 ± 1.6	38.6 ± 1.9	0.08 (−2.19 to 2.35)	0.93
TSL (°C)	30.9 ± 0.9	29.7 ± 1.1	1.28 (0.01–2.55)	0.05
CPT (°C)	5.5 ± 1.3	8.0 ± 1.6	2.50 (−4.40 to −0.62)	0.01
HPT (°C)	45.1 ± 0.6	44.1 ± 0.9	1.06 (0.07–2.05)	0.04
Detection-to-pain interval (ΔT)				
CDT-CPT (°C)	16.2 ± 2.5	12.7 ± 2.5	3.47 (0.25–6.68)	0.04
HPT-WDT (°C)	6.4 ± 1.1	5.6 ± 0.9	0.85 (−0.40–2.10)	0.16
MPT (mN)	51.8 ± 10.9	51.6 ± 14.3	0.17 (−16.18–16.54)	0.98
MPS (NRS)	2.0 ± 0.5	2.4 ± 0.7	0.46 (−1.25–0.33)	0.22
TSP (ΔNRS)	0.7 ± 0.3	1.3 ± 0.5	0.58 (−1.1 to −0.06)	0.03

Data of 6 locations (depicted in Fig. 1) are averages. ΔT, delta temperature, the difference between the 2 thresholds. A *P*-value of < 0.05 was considered statistically significant.

CDT, cold detection threshold; CPT, cold pain threshold; HPT, heat pain threshold; MPT, mechanical pain threshold; MPS, mechanical pain sensitivity; NRS, numerical rating scale; TSP, Temporal Summation of Pain; TSL, thermal sensory limen; WDT, warm detection threshold.

**Figure 3.**
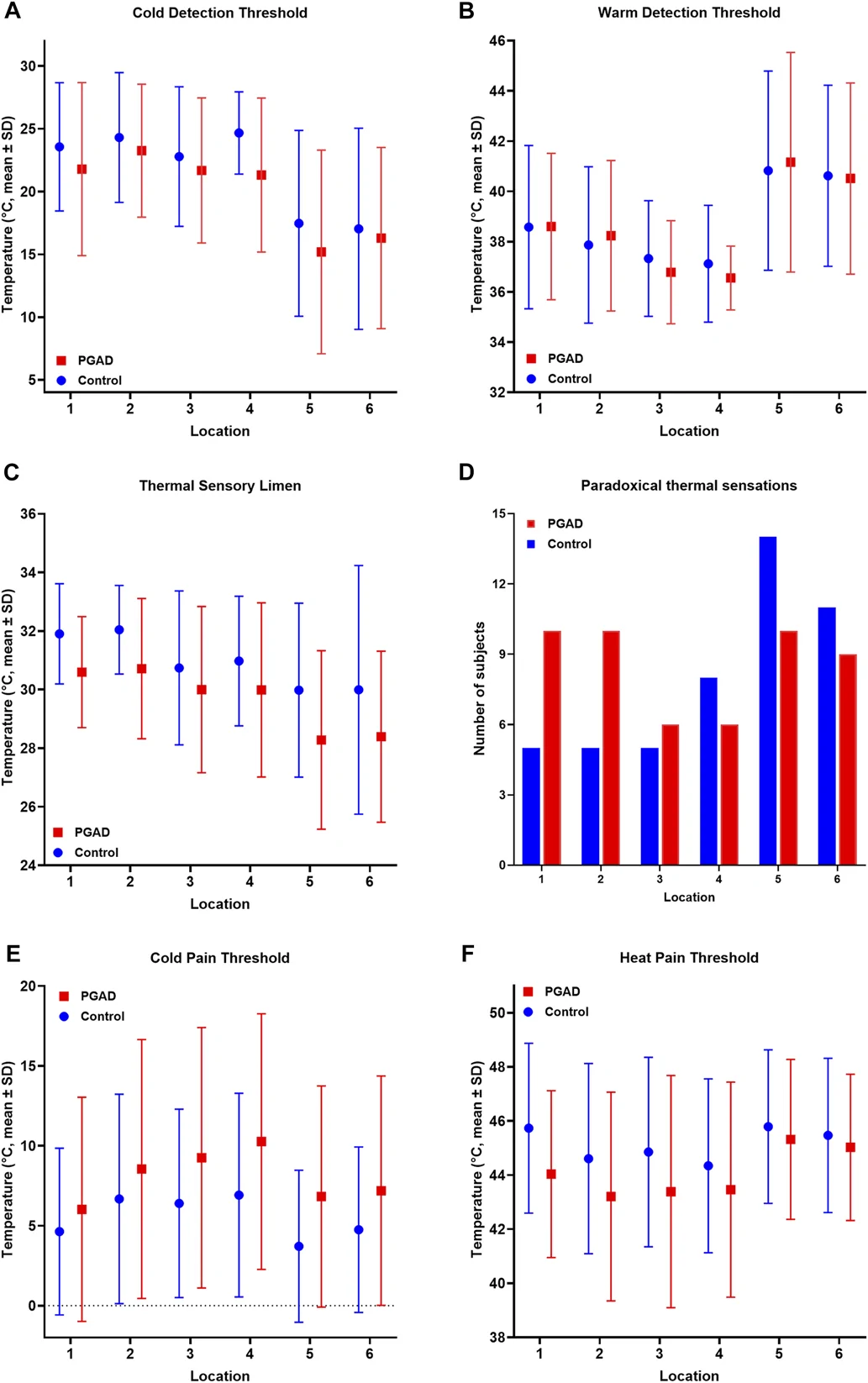
Results of thermal tests of quantitative sensory testing for genital locations of PGAD participants (red squares) and controls (blue circles). For panels A–C and E–F, average threshold temperature ± SD are plotted. For panel D, the number of subjects who report a paradoxical thermal sensation on each location is visualized. Locations are marked 1 to 6, corresponding to the locations depicted in Figure 1. The overall group difference was not statistically significant for panel A, *P* = 0.40; panel B, *P* = 0.93; and panel D, *P* > 0.99. For panel C, E, and F, there was overall statistically significant difference, respectively, *P* = 0.05, *P* = 0.01, and *P* = 0.04. PGAD, persistent genital arousal disorder.

Cold pain thresholds were significantly different in PGAD participants (8.0 ± 1.6°C) compared with controls (5.5 ± 1.3°C, *P* = 0.01) and heat pain thresholds as well (44.1 ± 0.9°C vs 45.1 ± 0.6°C, *P* = 0.04) (Fig. 3E–F). These findings indicate cold and heat hyperalgesia in PGAD. The detection-to-pain interval for cold stimuli was significantly smaller in PGAD participants (12.7 ± 2.5°C) than in controls (16.2 ± 2.5°C, *P* = 0.04), whereas the interval for heat stimuli did not differ significantly (*P* = 0.16) (Table 3).

Mechanical pain thresholds did not differ between PGAD participants and controls (51.6 ± 14.3 mN vs 51.8 ± 10.9 mN, *P* = 0.98). Mechanical pain sensitivity scores were numerically higher in PGAD participants but did not differ significantly (2.4 ± 0.7 vs 2.0 ± 0.5, *P* = 0.22) (Table 3 and Fig. 4).

**Figure 4.**
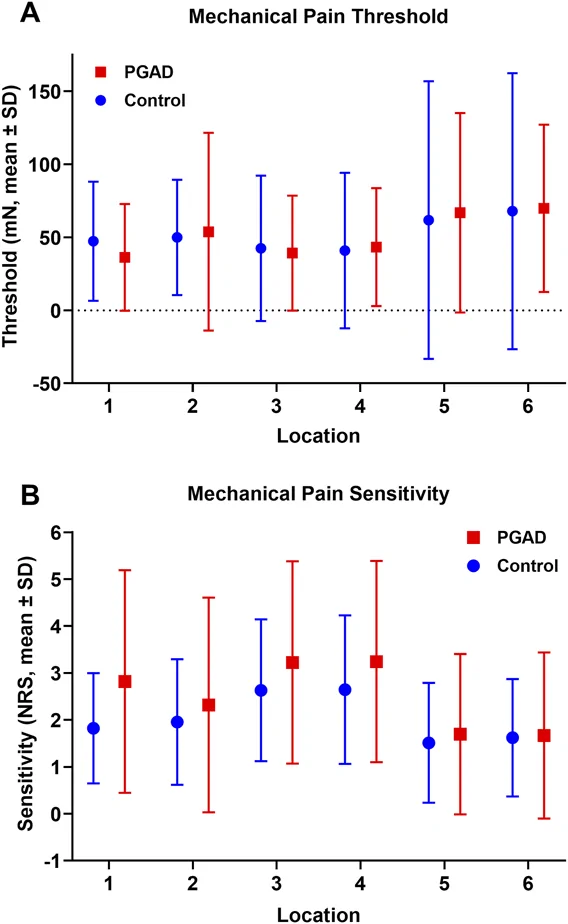
MPT and MPS scores for genital testing in PGAD participants (red squares) and controls (blue circles). Locations are marked 1 to 6, corresponding to the locations depicted in Figure 1. For panel A, the *y*-axis depicts the average pain threshold in millinewtons. In panel B, the *y*-axis marks the average pain score. In both panels, error bars represent SDs. There is no statistically significant difference in MPT, *P* = 0.98, and MPS, *P* = 0.22. MPS, mechanical pain sensitivity; MPT, mechanical pain thresholds; PGAD, persistent genital arousal disorder.

Dynamic mechanical allodynia in the genital region was observed more frequently in PGAD participants (33%) than in controls (13%), although nonsignificant (*P* = 0.12). Location-specific subanalysis revealed significant group differences at locations 4 and 6, with more severe allodynia in PGAD participants (Fig. 5A). Among participants who exhibited allodynia, the median number of affected locations was higher among the 10 PGAD participants (median 4, IQR 4) than with the 4 controls (median 1.5, IQR 2.5), although this difference was not statistically significant (*P* = 0.07). Finally, mean pain intensity ratings to mechanical stimuli on 6 locations were higher in PGAD participants than in controls (3.7 ± 0.8 vs 1.4 ± 0.5, *P* < 0.0001) (Fig. 5B).

**Figure 5.**
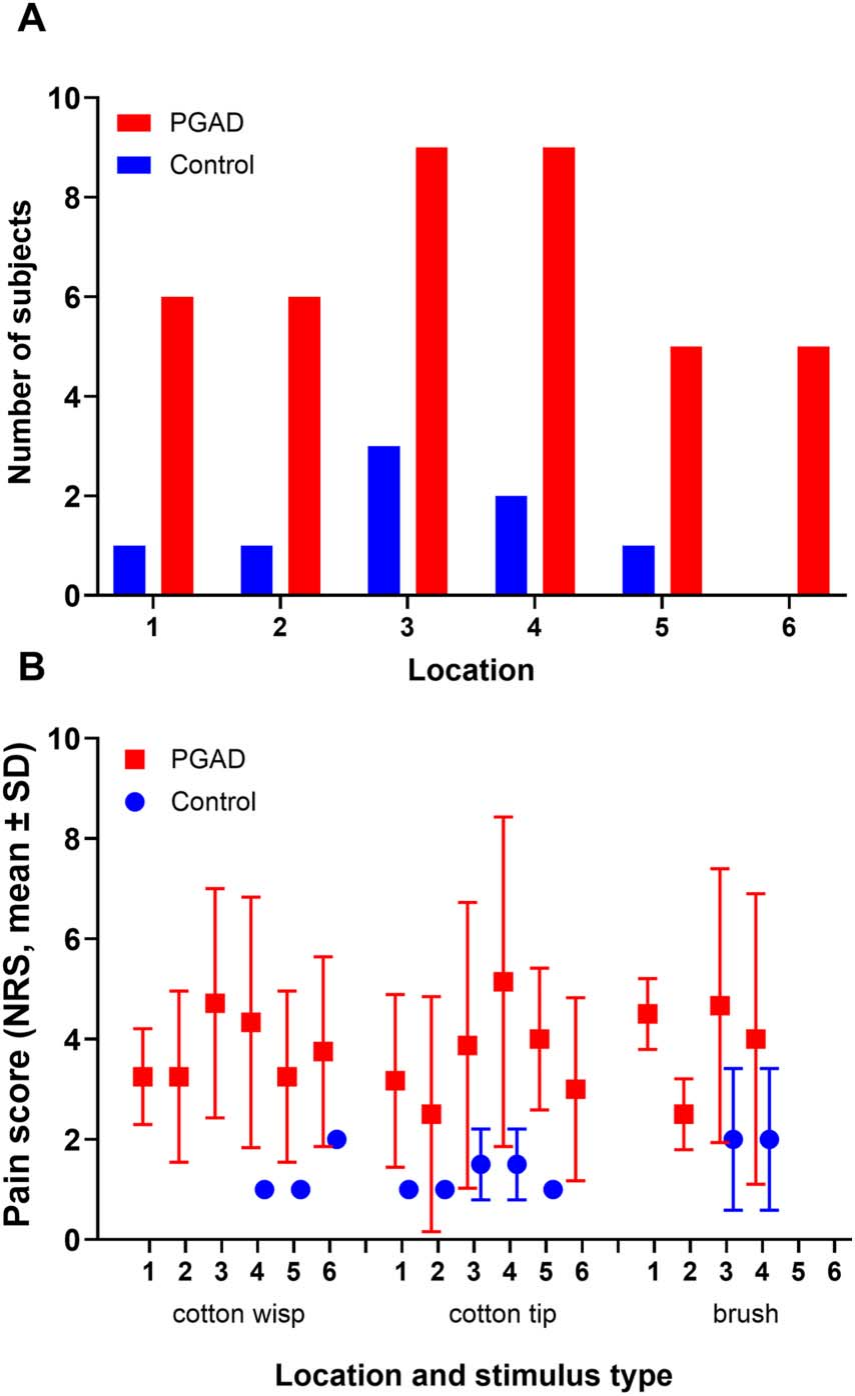
(A) Prevalence of dynamic mechanical allodynia across 6 locations in PGAD participants (red squares) and controls (blue circles). Locations are marked 1 to 6, corresponding to the locations depicted in Figure 1. The overall group difference was not statistically significant, *P* = 0.12. (B) Mean NRS ± SD pain ratings for mechanical stimuli across 6 locations in PGAD participants (red squares) and controls (blue circles). Mean NRS was significantly higher in PGAD vs controls *P* < 0.0001. NRS, numerical rating scale; PGAD, persistent genital arousal disorder.

The wind-up response, expressed as the change in pain intensity (NRS), differed significantly between groups. Participants persistent genital arousal disorder showed a greater increase in mean pain ratings during repeated stimulation compared with controls (1.32 ± 0.48 vs 0.73 ± 0.31, *P* = 0.03, Fig. 6), consistent with enhanced temporal summation of pain.

**Figure 6.**
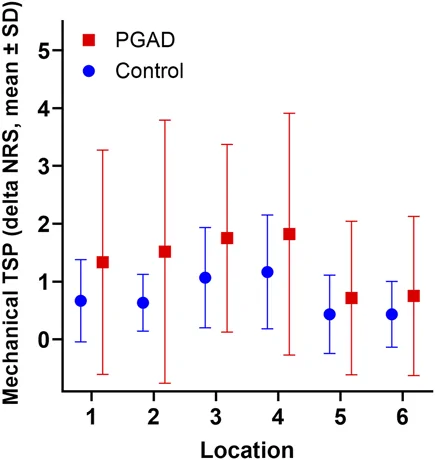
Mechanical TSP across tested genital locations. Data are ΔNRS ± SD (ie, pain rating after 10 stimuli minus pain rating after 1 mechanical stimulus): PGAD participants (red squares) and controls (blue circles). Data are presented per location as depicted in Figure 1. The overall groups difference was statistically significant (*P* = 0.03). NRS, numerical rating scale; PGAD, persistent genital arousal disorder; TSP, Temporal Summation of Pain.

One PGAD participant experienced mild redness of the genital skin adjacent to the testing site accompanied by temporary hyperalgesia following thermal testing (adverse event). Symptoms resolved spontaneously within 48 hours without sequelae.

### 3.4. Conditioned pain modulation

Conditioned pain modulation was evaluated in 28 PGAD participants and 29 controls. In 3 participants, measurements could not be obtained due to unreliable responses. The mean peak pain score for the test stimulus was 60.0 ± 7.6 mm (mean ± SD, range 49–77 mm), in PGAD participants and 58.0 ± 7.7 mm (43–74 mm) in controls. Cold-water conditioning elicited mean pain ratings of 3.7 ± 2.8 and 3.3 ± 3.1 (NRS), respectively.

During conditioning, the average peak pain score decreased to 45.0 ± 16.7 mm (13–71 mm) in PGAD participants and 49.0 ± 15.2 mm (26–82 mm) in controls. The relative average CPM in our cohort was 25.9 ± 37.3% (−16% to 73%) in PGAD participants and 15.7 ± 22.3% (−32% to 57%) in controls, with no significant between-group difference. Using a predefined cutoff of 12% inhibition, ineffective CPM was observed in 38.5% PGAD participants and 41.4% of controls (*P* > 0.05).^17^

### 3.5. Corneal confocal microscopy

Corneal confocal microscopy was performed in 29 PGAD participants and 29 controls, measurements failed in 2 participants due to technical problems. Because measurements from the left and right eye were comparable, the results were averaged. The median CNFD and corneal nerve fiber length values were 18.7 mm/mm^2^ and 11.4 mm, respectively, in PGAD participants. Abnormal CNFD values were observed in 7 PGAD participants (24%) and 6 controls (21%), abnormal CNBD in 14 PGAD participants (48%) and 14 controls (59%), and abnormal corneal nerve fiber length in 22 PGAD participants (76%) and 17 controls (59%). No statistically significant group differences were observed (*P* > 0.05).

## 4. Discussion

In this exploratory case–control study, we performed detailed sensory testing in women with PGAD and compared the findings with healthy controls. Overall, our study confirms that QST is feasible for characterizing somatosensory function in women with PGAD. Our results suggest that a subset of women with PGAD shows a sensory profile compatible with localized genital hypersensitivity and enhanced temporal summation.

Genital QST revealed altered thermal pain sensitivity consistent with thermal hyperalgesia, in the PGAD group compared with controls. Other genital sensory measures did not differ significantly between groups. Previous studies have shown that genital sensory testing can detect abnormalities in women with genital or sexual complaints. Women with arousal or orgasmic dysfunction have demonstrated abnormal genital sensory thresholds compared with normative values, suggesting a neurophysiological contribution.^8^ Normative studies have shown that genital sensory sensitivity varies by anatomical site and declines with age, underscoring the importance of physiological variation when interpreting genital sensory data.^27^ Altered genital sensation has been reported in women with chronic disease or pelvic disorders, although its clinical relevance remains uncertain due to limited statistical power.^1^

Our findings extend previous literature by demonstrating that sensory abnormalities in PGAD can be detected using standardized QST. The predominantly regional pattern of abnormalities supports a localized alteration in genital sensory processing rather than a generalized somatosensory disorder, possibly involving pudendal nerve dysfunction or hyperexcitability. Although PTS were uncommon and did not differ significantly between groups, they occurred more frequently in PGAD participants and may indicate altered thermal sensory integration in a subset of patients. Mechanical allodynia was also more prevalent in PGAD participants, who showed higher pain ratings to normally nonpainful stimulation. Although prevalence differences did not reach statistical significance, higher NRS ratings and more affected genital sites indicate enhanced genital sensory responsiveness in PGAD. This aligns with a study in another pelvic pain condition, in which allodynia has been interpreted as neuropathy-like sensory involvement.^11^ The virtual absence of allodynia at the hand control site supports that sensory abnormalities observed in PGAD are predominantly localized rather than generalized.

Enhanced temporal summation of mechanical pain was observed in the PGAD group, indicating increased facilitation of nociceptive input during repeated stimulation. This may reflect central amplification of genital sensory input and could contribute to the persistence and intrusiveness of PGAD symptoms. Although these findings do not establish central sensitization, they suggest that changes in central nociceptive processing may coexist with localized genital sensory abnormalities in a subset of patients.

Because this was an exploratory study, genital QST was performed at predefined standardized sites to maximize reproducibility. Given heterogeneous symptom distribution in PGAD, this may have reduced sensitivity to detect local sensory abnormalities. For mostly ethical reasons, QST was not performed directly on the clitoris, which is often considered the most sensitive and affected site in women with PGAD. Future studies should incorporate symptom-guided QST to determine whether abnormalities are greatest at sites of peak symptom expression. In addition, no dedicated clinical sensory examination of the genital regions was performed. Quantitative sensory testing findings could not be directly related to clinically demonstrable neurological sensory abnormalities, which should be addressed in future studies combining QST with detailed neurological examinations.

Contrary to our hypothesis, CPM did not differ significantly between groups. Although nearly 40% of PGAD participants showed ineffective CPM, this proportion was similar in controls. These findings suggest that impaired descending pain inhibition is not a consistent feature of PGAD. Instead, interindividual variability may reflect phenotypic heterogeneity, with only a subset of patients showing impaired endogenous pain control. Similar CPM variability has been reported across neuropathic pain syndromes and may indicate mechanistically distinct subtypes.^6^ In our cohort, descending pain inhibitory function appeared preserved in many women with PGAD. Future studies should investigate the possibility of a more nociplastic pain phenotype in patients with PGAD.

Corneal confocal microscopy provided additional, albeit mixed, evidence for systemic peripheral small fiber involvement. Although not all CCM parameters differed significantly, reduced corneal nerve fiber measures were observed in a proportion of PGAD participants, consistent with small fiber pathology. This is relevant because small fiber neuropathy has been implicated in chronic pain disorders, including fibromyalgia and painful diabetic neuropathy.^7,25^ Similar CCM abnormalities in PGAD would support a systemic peripheral neuropathic contribution.^4^ However, overlap with control values indicates that these abnormalities are not universal, further underscoring the heterogeneity of PGAD.

The sensory abnormalities observed in PGAD should be considered within the broader spectrum of chronic pelvic pain disorders, including vulvodynia and bladder pain syndrome. Similarly, PGAD has features such as hypersensitivity, dysesthesia, and pain evoked by non-noxious stimuli. Quantitative sensory testing findings in pelvic pain populations suggest that altered sensory processing may also contribute to PGAD.^16,18,22^ The absence of consistent CPM abnormalities in our cohort argue against a uniform nociplastic pain mechanism; this deserves further study in larger cohorts allowing reliable detection of phenotypic subgroups.

Overall, our findings indicate involvement of peripheral and central nervous system abnormalities in PGAD, with a subset of patients exhibiting a localized sensory phenotype with altered genital nociceptive processing. Variability across measures suggests that PGAD does not represent a single mechanistic entity. Instead, distinct phenotypes may exist, ranging from localized peripheral neuropathy to widespread central sensory amplification. The most consistent abnormalities involved genital sensory processing, with altered thermal pain sensitivity and enhanced temporal summation suggesting localized nociceptive alterations. This distinction is clinically relevant, as identifying these patterns supports tailored treatment strategies. Patients with a peripheral phenotype may benefit from interventions targeting the pudendal nerve or genital sensory pathways, such as topical therapies or nerve blocks. By contrast, patients with central pain dysregulation may be better candidates for systemic pharmacological treatments, neuromodulation, or psychologically informed interventions such as cognitive behavioral therapy. Enhanced temporal summation may reflect increased nociceptive facilitation but does not confirm central sensitization in PGAD. It suggests that altered central processing may coexist with localized peripheral sensory abnormalities, emphasizing the importance of objective phenotyping for individualized care.

This study has several strengths. We combined genital and extragenital QST, CPM, and CCM in a single protocol, enabling assessment of multiple sensory domains. This multimodal approach provides a broader view of somatosensory function than individual methods alone. Several limitations should also be acknowledged. First, normative values for genital DFNS-QST are unavailable, limiting interpretation of absolute sensory thresholds. Although normative data have been reported, used methods differ from the current QST protocol and are therefore not directly comparable. Nevertheless, inclusion of a well-matched healthy control group provided relevant reference. Second, sample size was based on feasibility rather than formal calculations, and the study may have been underpowered for exploratory outcomes. Negative findings should be interpreted cautiously, and the results should be considered hypothesis generating rather than confirmatory, particularly given risk of type I errors. Third, menstrual cycle and hormonal status of participants were not assessed. Although hormonal effects on pain sensitivity are generally modest and many participants were postmenopausal, future studies could examine hormonal influence on sensory function and experimental pain sensitivity. Finally, coexisting nociplastic pain conditions and other causes of peripheral neuropathy were not systematically assessed or excluded. Therefore, it remains unclear to what extent observed alterations are specifically attributable to PGAD or reflect mixed-complex nociplastic conditions. Despite these limitations, this study provides a multimodal sensory characterization of women with PGAD using genital QST, CPM, and CCM. The findings support sensory heterogeneity within PGAD and provide a foundation for future studies aimed at identifying mechanistic phenotypes to develop targeted treatment strategies.

## Disclosures

Author M. Niesters received consultancy/speaker fees from Grünenthal GmbH and Amgen Inc. Author A. Dahan received consultancy/speaker fees from Enalare Therapeutics, Princeton, NJ, Trevena Inc., Chesterbrook, PA, and Zmi Pharma Inc., Carlsbad, CA, and an award from the US Food and Drug Administration, Silver Spring, Maryland. All other authors report no competing interests.
